# Detection of *Mycobacterium tuberculosis* bacilli in bio-aerosols from untreated TB patients

**DOI:** 10.12688/gatesopenres.12758.2

**Published:** 2018-06-08

**Authors:** Benjamin Patterson, Carl Morrow, Vinayak Singh, Atica Moosa, Melitta Gqada, Jeremy Woodward, Valerie Mizrahi, Wayne Bryden, Charles Call, Shwetak Patel, Digby Warner, Robin Wood

**Affiliations:** 1 Division of Infectious Diseases, Department of Medicine, Columbia University, College of Physicians and Surgeons, New York, NY, USA; 2 Institute of Infectious Disease and Molecular Medicine (IDM), Faculty of Health Sciences, University of Cape Town, Cape Town, South Africa; 3 Desmond Tutu HIV Centre,Institute of Infectious Disease and Molecular Medicine (IDM), University of Cape Town, Cape Town, South Africa; 4 MRC/NHLS/UCT Molecular Mycobacteriology Research Unit & DST/NRF Centre of Excellence for Biomedical TB Research, Department of Pathology, Faculty of Health Sciences, University of Cape Town, Cape Town, South Africa; 5 Department of Integrative Biomedical Sciences, Faculty of Health Sciences, University of Cape Town, Cape Town, South Africa; 6 Zeteo Tech LLC, Ellicott City, Maryland, USA; 7 Computer Science and Engineering, Electrical Engineering DUB group, University of Washington, Seattle, USA

**Keywords:** respiratory aerosol sampling chamber, bio-aerosol, viable impaction, ddPCR assay, RD9

## Abstract

**Background**: Tuberculosis (TB) is predominantly an airborne disease. However, quantitative and qualitative analysis of bio-aerosols containing the aetiological agent, *Mycobacterium tuberculosis (Mtb)*, has proven very challenging. Our objective is to sample bio-aerosols from newly diagnosed TB patients for detection and enumeration of *Mtb *bacilli.

**Methods**: We monitored each of 35 newly diagnosed, GeneXpert sputum-positive, TB patients during 1 hour confinement in a custom-built Respiratory Aerosol Sampling Chamber (RASC). The RASC (a small clean-room of 1.4m ) incorporates aerodynamic particle size detection, viable and non-viable sampling devices, real-time CO _2_ monitoring, and cough sound-recording. Microbiological culture and droplet digital polymerase chain reaction (ddPCR) were used to detect *Mtb *in each of the bio-aerosol collection devices.

**Results**: *Mtb* was detected in 27/35 (77.1%) of aerosol samples; 15/35 (42.8%) samples were positive by mycobacterial culture and 25/27 (92.96%) were positive by ddPCR. Culturability of collected bacilli was not predicted by radiographic evidence of pulmonary cavitation, sputum smear positivity. A correlation was found between cough rate and culturable bioaerosol. *Mtb* was detected on all viable cascade impactor stages with a peak at aerosol sizes 2.0-3.5μm. This suggests a median of 0.09 CFU/litre of exhaled air (IQR: 0.07 to 0.3 CFU/l) for the aerosol culture positives and an estimated median concentration of 4.5x10 CFU/ml (IQR: 2.9x10 -5.6x10 ) of exhaled particulate bio-aerosol.

**Conclusions**: *Mtb* was identified in bio-aerosols exhaled by the majority of untreated TB patients using the RASC. Molecular detection was more sensitive than mycobacterial culture on solid media, suggesting that further studies are required to determine whether this reflects a significant proportion of differentially detectable bacilli in these samples.

## Introduction

Tuberculosis (TB) has surpassed HIV/AIDS as a global killer with more than 4000 daily deaths ^1^. The rate of decline in incidence remains inadequate at a reported 1.5% per annum ^1^ and it is unlikely that treatment alone will significantly reduce the burden of disease ^2^. In communities with highly prevalent HIV, *Mycobacterium tuberculosis (Mtb)* genotyping studies have found that recent transmission, rather than reactivation, accounts for the majority (54%) of incident TB cases ^3^. Therefore, interruption of transmission would likely have a rapid, measurable impact on TB incidence. The physical process of TB transmission remains poorly understood and the application of new technologies to elucidate key events in infectious aerosol production, release, and inhalation, has been slow.

The capacity for airborne transmission of *Mtb* bacilli was first demonstrated in an elegant series of experiments by Richard Riley and colleagues nearly seventy years ago ^4^. Venting exhaled air from pulmonary TB patients over a guinea pig facility resulted in infection of the animals leading to the concept of infectious ‘quanta’ (the dose of infectious air required to cause an infection). Notably, these pioneering studies indicated that quanta production was extremely infrequent and definitively attributable to only a small minority of patients. Furthermore, the quantitative relationship between airborne infectious particles and quanta remains unclear.

Empirical studies to characterise airborne infectious particles have been sparse. Two major difficulties plaguing investigation are the purportedly low concentrations of naturally produced *Mtb* particles, and the complication of environmental and patient-derived bacterial and fungal contamination of airborne samples ^5^. There have nonetheless been a number of attempts at airborne detection. ^6^,^13^.

Of particular interest, a proof of concept study ^12^ and subsequent feasibility study in Uganda ^14^ sampled cough-generated aerosols from pulmonary TB patients. Coughing directly into a sampling chamber equipped with two viable cascade impactors resulted in positive cultures from more than a quarter of participants despite their having received 1–6 days of chemotherapy. A follow-up work employing the same apparatus found that participants with higher aerosol bacillary loads could be linked to greater household transmission rates ^14^ and development of disease ^15^ findings which suggest that quantitative airborne sampling may serve as a clinical relevant measure of infectivity.

In this study, we aimed to gain further insight into the airborne phase of TB and establish the bacillary concentration in exhaled bio-aerosols. We used the respiratory aerosol sampling chamber (RASC) ^16^, a novel apparatus designed to optimise patient-derived aerosol sampling, to isolate and accumulate respirable aerosol from a single patient. Environmental sampling detects the *Mtb* present after a period of ageing in the chamber air. The resulting ‘dried residua’ ^17^, formed from larger respiratory droplets, are predicted to mimic more closely the putative infectious particle.

## Methods

### Ethical statement

Ethics approval was obtained from the University of Cape Town Faculty of Health Sciences Human Research Ethics Committee (HREC/REF: 680/2013). Written informed consent for publication of the participants details was obtained from the participants. Sampling took place on the same day as treatment initiation with a typical delay of 1–2 hours to complete the study protocol.

### Subject recruitment

Participants who had tested positive for drug-sensitive pulmonary TB by GeneXpert were recruited prior to initiation of chemotherapy from a peri-urban township 40km south of Cape Town

Baseline patient data were collected from the clinical records and a chest X-ray was taken approximately seven days after the start of treatment. The presence of lung cavitation was scored by one of the authors (BP) based on the chest X-ray and this score was compared to a radiologist report for agreement.

### Respiratory Aerosol Sampling Chamber protocol

The Respiratory Aerosol Sampling Chamber (RASC) has previously been described in detail ^16^. The RASC consists of a small personal clean space (1.4 m ) in which a participant is seated and engages passively in an exhaled air sampling protocol. Approximately an hour is spent in the chamber following the phases outlined in Wood *et al*. ^16^ Briefly, the chamber is sealed and an air purge phase is performed entraining ambient air through high-efficiency particulate arrestance (HEPA) filters for a period of 10 minutes. This is followed by a participant-driven contamination phase in which the chamber is isolated from the external environment and the proportion of exhaled air allowed to rise to a 10% threshold defined by a chamber CO _2_ concentration of 4,000 ppm above the ambient level (based on an assumed exhaled air CO _2_ concentration of 40,000 ppm). If the target is not reached after 30 minutes have elapsed, the sampling phase is started at a lower exhaled air proportion. After sampling, the chamber is again purged to remove residual *Mtb* from the air.

Contamination of the sampling chamber was driven primarily by tidal breathing in addition to spontaneous coughing or sneezing. Particles and organisms derived from sources other than breath were minimised by the participant wearing a full-body DuPont Tyvek suit during sampling and an initial purge phase to minimise ambient contamination. Drawing the chamber air over a range of devices allowed mycobacterial detection by microbiological culture or molecular quantitation of genome equivalents.

### Particle size measurement

Aerosolized particles were monitored from the final minute of the purge phase and throughout the remainder of the experimental protocol via an aerodynamic particle sizer (APS Model 3321, TSI, Shoreview, MN USA).

***Sound recording.*** Sounds from the inside of the sampling chamber were recorded by microphone and stored as 44.1 KHz 16-bit WAV files using a custom-built recorder application. The files were securely transmitted to a server where automated cough sound analysis can occur. The cough sound analysis divides the input recording into multiple segments of time, and a machine learning algorithm classifies each segment of time as either a cough or not a cough, using characteristics of the signal at that moment in time such as the overall energy within the signal, the distribution of energy across frequencies and the amount of change in energy within the signal within that segment. These classifications are then merged together in order to identify longer segments in time that are continuously cough or non-cough segments, which are then used to identify periods of coughing. This analysis was used to determine cough frequency and cough length for each participant.

### Particle capture for microbiological analysis

The sampling phase utilised a six-stage viable Andersen Impactor (Model 10830-EPD, Thermo Scientific, USA) which allowed physical separation of aerosolized particles by size, based on the principle of inertial impaction. These captured particles are incubated to ascertain the number of Mtb bacilli released. The impactor sampled chamber air at a rate of 28 l/min for 10 minutes. Each impactor stage contained a glass Petri dish containing solid Middlebrook 7H10 medium further described below. For participants 14 to 35, a 0.2 µm polycarbonate filter (Sterlitech Corporation, WA USA) of 47 mm diameter was placed on the agar plate abutting the edge, and subsequently removed and analysed using droplet digital PCR (ddPCR).

Direct capture took place using a 0.4 µm microporous polycarbonate filter (Sterlitech Corporation, WA USA) positioned above the participant in an open-faced mount with a 20 l/min flow rate run for 10 minutes. The filter was cut with one half analysed by the microbiological culture method and the other half by ddPCR. A gel filter (Model 12602-37-ALK, Sartorius, Goettingen, Germany) was similarly positioned and run at a flow rate of 20 l/min for 10 minutes. A 0.4 µm polycarbonate filter was placed in-line and downstream of the gel filter. An open-faced, polyester felt filter of 47mm diameter and 1.0μm pore size (American Felt and Filter Company, New Windsor, New York; Lockheed Martin, Alexandria, VA, USA) was used to sample at a high flow rate (approx. 300 l/min) for 10 mins at the end of the experiment and was analysed by ddPCR (see below).

### Particle capture for imaging

A Dekati three-stage impactor (PM10, Dekati, Kangasala, Finland) sampling at 30 l/min for ten minutes was used to separate respired particles according to size onto uncoated aluminium foil discs. Assuming a particle density of 1 g/cm3, the three stages collect particles in the size ranges: >14.1 μm, 14.1 μm - 3.5 μm and 3.5 μm - 1.4 μm respectively. A 0.4 µm polycarbonate filter was placed at the outflow of this impactor to capture aerosols of less than 1.4 µm.

The foil discs were air-dried and sterilised by UV-irradiation before imaging, uncoated, by scanning electron microscopy (Zeiss/Leo 1450, ZEISS, Oberkochen, Germany) in secondary electron mode at 10 kv.

### Quantification of microbiological specimens

For an individual inside the RASC, the ratio of exhaled air volume to chamber volume is equal to the ratio of excess CO _2_ (measured CO _2_ less ambient CO _2_) to the CO _2_ in exhaled breath (approx. 40,000 ppm). Continuous CO _2_ monitoring therefore allowed a close approximation of the proportion of exhaled air volume for each participant in the RASC at any given time. The sampled exhaled air volume was the product of this proportion and the air volume sampled by each detection device. Concentrations of colony forming units (CFU) by unit volume of exhaled air could then be established for any of the sampling devices.

[CO2]excess=[CO2]measured−[CO2]ambient

$Exhaled\;Air\;Volume=\frac{[CO2]excess}{[CO2]exhaled}\times Device\;Flow\;Rate\times Device\;Sampling\;Time$

$CFU\;per\;unit\;Volume\;of\;Air=\frac{CFU\;count}{Exhaled\;Air\;Volume}$

Simultaneous measurement of the particle content of the chamber air at the point of microbiological sampling allowed calculation of an aerosol volume per unit volume of air sampled. From these two measures, an approximate *Mtb* CFU concentration by volume of bio-aerosol was determined (aerosol geometry assumed to be spherical).

$\frac{Bioaerosol\;Volume}{Sv\;Chamber\;Air}\left(single\;size\;bin\right)=\frac{\left(\frac{4}{3}\pi \;{\left(bin\;size/2\right)}^{3}\times particle\;count\right)}{5\times 10*}$

$\frac{Bioaerosol\;Volume}{Sv\;Chamber\;Air}\left(Andersen\;Stage\right)=\;\sum \frac{Bioaerosol\;Volume}{Sv\;Chamber\;Air}\;\left(corresponding\;size\;bins\right)$

$CFU\;per\;Volume\;Bioaerosol\;\left(Andersen\;Stage\right)=\frac{CFU\;count\;at\;stage}{Sv\;Chamber\;Air}÷\frac{Bioaerosol\;Volume}{Sv\;Chamber\;Air}\;\left(Andersen\;Stage\right)$

Sv = Volume Sampled

*APS sampled at rate of 5L/min; Andersen sampling over 10 minutes

***Microbiological detection methods***

**Culture**

Andersen Impactor plates were unloaded in a biosafety cabinet. Filters analysed by culture were placed face-up on solid Middlebrook 7H10 agar supplemented with Glycerol, OADC, and **0.05%** Tween80, PANTA (BD) antibiotic mixture and incubated at 37°C for 4–6 weeks. The number of CFU consistent with expected *Mtb* colony morphology and rate of formation *in vitro* was recorded, and genomic DNA extracted for PCR confirmation using primers RD9F (5’-gtgtaggtcagccccatcc-3’), RD9R (5’-gctaccctcgaccaagtgtt-3’) and RD9Int (5’gctaccctcgaccaagtgtt-3’) using a protocol developed elsewhere. ^18^.

**Protocol for RD9 confirmation of Mtb Colonies**

20 μl reactions containing 2 µl of template DNA are set up with 1x reaction buffer, 200 μM of each dNTP, 0.5–1.0 μM of each primer, 1.5 mM MgCl _2_, 1x GC-rich solution, and 2U/50 μl of DNA polymerase. Thermal cycler parameters used for DNA amplification are as follows: denature at 95°C for 5 min, followed by 40 cycles of denaturation (94°c for 30s), annealing (65°C for 1min), and extension (72°C for 10min), and a final extension at 72°C for 7 min.

**Droplet digital PCR (ddPCR)**

Filters were removed from the RASC and transported in 50 ml Falcon tubes. The specimen was processed by vortexing in sterile PBS + 0.05% Tween 80 and centrifuged (3750 rpm for 15 minutes) to harvest the pellet which was lysed for DNA extraction and purification. Quantitative analysis of the RD9 region used a modified protocol and the QIAamp DNA Mini Kit (Qiagen). Primers (RD9/qRTF 5’-tgagtggcgatggtcaacac-3’ and RD9/qRTR 5’-gatggcgttcggaaagaaac-3’) and TaqMan minor groove binder (MGB) probe (RD9/probe 5’-actacgcggcttagtg-3’) were designed using Primer Express software (version 3.0.1). TaqMan MGB probe homologous to the RD9 gene was labelled with 6-carboxyfluorescein (FAM). The ddPCR reaction set-up and the run were performed as described previously ^19^.

An evaluable result for TB DNA was assigned when the following conditions were satisfied: (i) the total number of droplets read in the well was greater than 10,000; (ii) positive droplets possessed a fluorescence intensity above a threshold of 3500; (iii) minimal numbers of intermediate droplets (“rain”) were observed between positive and negative values; and (iv) the observed droplet distribution was consistent with a subpopulation of the positive control which comprised known concentrations of genomic DNA extracted from *Mtb* H37Rv.

### Statistical analysis

Participants were divided into groups according to positive or negative airborne culture. Groups were then compared with unadjusted analyses using Fisher’s exact tests for categorical variables and Wilcoxon rank sum tests for continuous variables. Airborne particles and cough data were investigated using Pearson’s correlation. Statistical analyses were performed using R Core Team (2015) ^20^.

## Results

### Baseline characteristics and microbiological results

A total of 35 participants were recruited for this study ( Table 1), all of whom had drug-sensitive pulmonary TB defined by a positive GeneXpert sputum. The mean age of the participants was 33 years, of which 57.1% were men and 48.6 % were HIV positive.

**Table 1.** Baseline characteristics of RASC participants.

Participant	Age	Sex	HIV status (CD4)	Previous TB	Aerosol Culture (CFU)	Aerosol ddPCR	Chest XR Cavitation	Coughs per hour
1	30	M	Negative	N	Positive (11)	Positive	N	N/A
2	36	F	Positive (61)	Y	Positive (2)	Positive	N	20
3	45	M	Negative	N	Positive (3)	Positive	N	22
4	24	F	Positive (553)	Y	Positive (1)	Negative	N	63
5	42	F	Positive (602)	Y	Negative	N/A	Y	59
6	39	M	Positive (621)	N	Positive (2)	N/A	N	11
7	33	M	Negative	N	Positive (3)	Positive	Y	25
8	40	F	Positive (39)	Y	Positive (3)	Positive	N	27
9	24	M	Negative	Y	Negative	Positive	N	2
10	24	F	Positive (115)	N	Negative	Positive	N	51
11	29	M	Positive (128)	N	Negative	Positive	N	16
12	32	M	Negative	N	Positive (8)	Positive	Y	52
13	22	F	Positive (371)	N	Negative	Positive	N	71
14	53	M	Negative	Y	Negative	Positive	N	36
15	37	M	Positive (1)	N	Negative	Positive	Y	7
16	46	M	Positive (228)	Y	Positive (1)	Positive	N/A	12
17	28	F	Positive (211)	N	Negative	Positive	Y	14
18	38	M	Positive (66)	Y	Positive (2)	Positive	N	64
19	46	M	Negative	N	Negative	Positive	N	6
20	18	F	Negative	N	Negative	Positive	Y	7
21	33	M	Positive (99)	N	Negative	Positive	Y	30
22	26	F	Negative	N	Negative	Unevaluable	N	8
23	22	F	Negative	N	Negative	Unevaluable	N	5
24	33	F	Positive (43)	N	Negative	Unevaluable	N	12
25	28	M	Positive (63)	N	Negative	N/A	N	N/A
26	31	M	Negative	N	Positive (2)	Positive	Y	9
27	25	M	Negative	N	Positive (14)	Positive	Y	33
28	22	F	Negative	N	Positive (1)	Positive	Y	49
29	22	F	Positive (630)	Y	Negative	N/A	Y	0
30	62	M	Negative	N	Negative	Negative	N	3
31	22	M	Negative	Y	Negative	N/A	Y	15
32	36	M	Negative	N	Positive (2)	Positive	Y	13
33	38	F	Positive (N/A)	Y	Positive (4)	Positive	N/A	136
34	26	F	Negative	N	Negative	Positive	N	20
35	52	M	Negative	N	Negative	Positive	N	0

N/A = not tested

**Figure 1.**
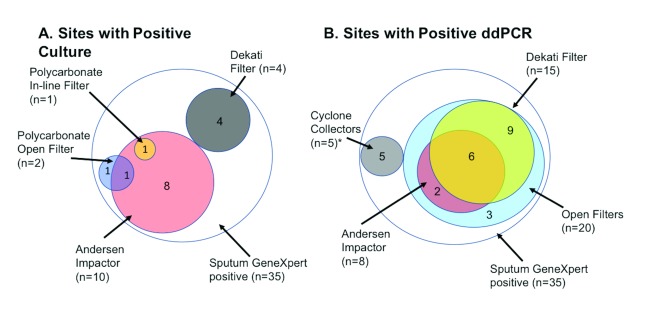
Euler Diagrams demonstrating successful detection sampling modalities for **A**. mycobacterial culture and **B**. ddPCR (25 positive out of 27 successfully tested). *Cyclone Collectors were added for the later participants. These included a NIOSH two-stage cyclone aerosol sampler (2 positive out of 4 participants sampled) and Coriolis µ biological air sampler (3 positive out of 3 participants sampled).

15 participants (42.9%) had a positive bio-aerosol mycobacterial culture, which was defined as one or more CFU detected on any of the sampling devices ( Figure 1A). 59 CFU exhibited the morphologies and growth rates characteristic of *Mtb* grown *in vitro* on solid media, and this was confirmed by RD9 genotype in 37 cases. For the other putative *Mtb* CFU, RD9 confirmation was not possible owing to fungal contamination. The median amongst the positives was 2.5 CFU with a range of 1–14. The greatest yield was with the viable Andersen cascade impactor which gave a median concentration of 0.09 CFU per litre of air sampled (IQR: 0.07 to 0.3 CFU/L). For the same device, the calculated median concentration of CFU in exhaled bio-aerosol was 4.5×10 CFU/ml (IQR: 2.9×10 –5.6×10 ).

Mean CFU per litre was 0.006 for the polycarbonate filters in the open-faced filter. The gel filter produced no positive results, whereas the downstream in-line polycarbonate filter produced a concentration of 0.003 CFU per litre of exhaled air. The concentration inferred from the Dekati outflow polycarbonate filter was 0.008 CFU per litre of exhaled air.

Samples from five of the participants were not tested by ddPCR, and a further three participants’ specimens were unevaluable. 25 participants (92.6%) out of 27 successfully tested had a positive ddPCR result from one or more of the sampling devices ( Figure 1B). Of all filters tested for all participants, 118/137 (86.1%) were positive for *Mtb*. By either method, *Mtb* was detected in 27 out of 35 participants (77.1%).

Production of a culturable bio-aerosol was not statistically associated with any of the recorded baseline characteristics, including cavitary disease on chest imaging and presence of a positive sputum smear whether treated as a binary (positive/negative) or continuous outcome. However, an association was observed between culturable bio-aerosol production and greater spontaneous cough frequency during the experimental protocol (see Table 2).

**Table 2.** Traditional predictors of infectiousness stratified by Aerosol Culture. Categorical variables were compared using Fisher exact tests and continuous variables using a Wilcoxon rank sum test.

		Aerosol Negative	Aerosol Positive	p-value
n		20	15	
Sputum smear (%)	Positive	10 (50.0)	9 (60.0)	0.807
Cavitary disease (%)	Positive	7 (35.0)	6 (46.2)	0.782
Cough count (median [IQR])		12 [5.5, 25]	26 [15, 51]	0.022

**Figure 2.**
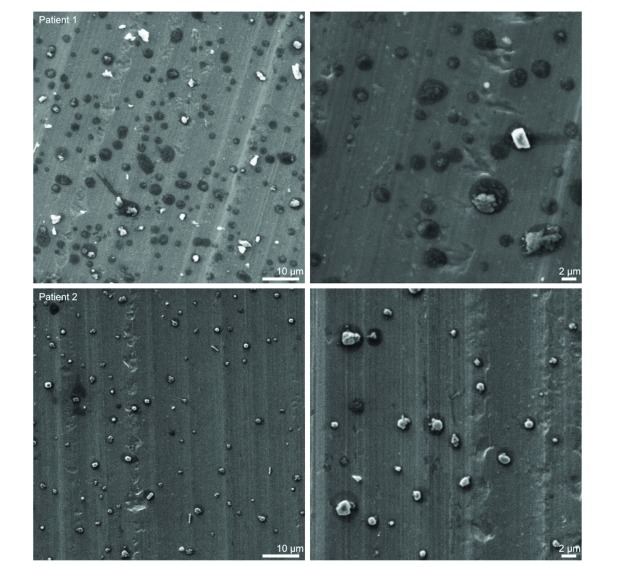
Respired particulate matter from two TB infected individuals captured on the lower plate (3.5 μm – 1.4 μm) of a Dekati three-stage impactor (PM10, Dekati, Kangasala, Finland) sampling at 30 l/m onto uncoated aluminium foil discs.

***Imaging of respired particulate matter.*** Representative images from two participants captured from SEM of the foil discs from the Dekati impactor are show in Figure 2. Numerous particles of variable morphology are shown which appear to comprise organic matter derived from patient lung or respiratory tract. Note the “halo” structures (dark shadows) surrounding each particle which may be indicative of droplet nuclei impaction.

### Particle analysis

**Figure 3.**
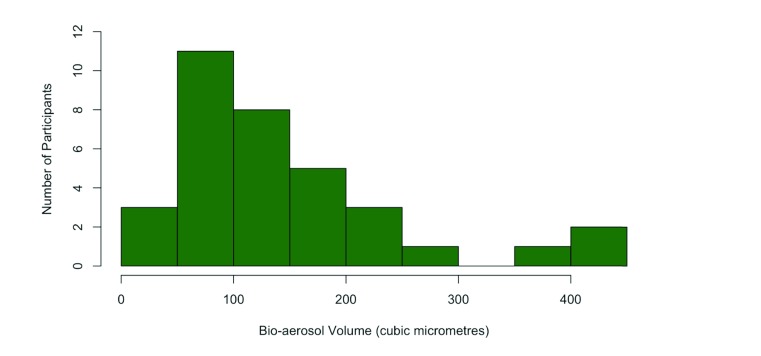
Histogram of bio-aerosol volume per litre of exhaled air for RASC participants.

Particle counts measured by the aerodynamic particle sizer (APS) were widely variable between participants. In general, counts gradually increased throughout the time spent in the chamber during the contamination phase. Comparison of particles among participants was performed by summing all the particles in the 1–5 µm range over a 5 minute period after 20 minutes of the contamination phase had elapsed. This result was divided by the number of litres sampled by the APS (25 L; 5 mins at flow rate of 5 l/min) yielding a 1–5 µm particle count per litre at the point of maximal contamination. Participant-derived particle counts were taken to be the difference between the figure at maximal contamination and the 1–5 µm particle count per litre concentration at the end of the purge phase. The median for the 35 pulmonary TB participants was 23.9 counts/l (IQR 14.8–47.7). A single outlier was excluded from this and the subsequent analysis owing to a markedly elevated particle count (>4 standard deviations from the mean), possibly due to environmental contamination of the chamber. Figure 3 shows a histogram of the 35 participants’ bio-aerosol production calculated as volume.

A strong correlation was observed between CO _2_ production rate and particle production with a Pearson’s correlation coefficient of 0.54 (95% CI 0.24 to 0.75; p<0.001) for the active TB participants. No relationship was elucidated between particle production and airborne culture when treated as a binary variable or quantitatively. No other significant correlations were inferred when comparing particle production with age, sex, HIV status, body mass index, severity of chest X-ray, presence of radiologically apparent cavitation or sputum smear status.

### Concentration and size distribution of bio-aerosol culture

**Figure 4.**
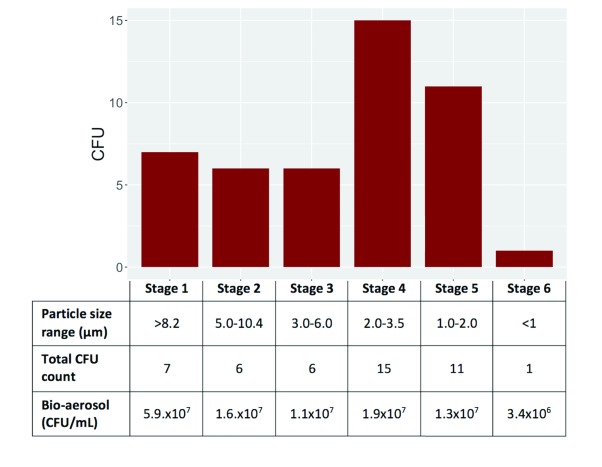
Histogram of total number of *M. tuberculosis* colony forming units in each of the 6 stages of Andersen impactors. The size range of collected particles and the calculated mean concentrations of CFUs per millilitre of captured bio-aerosol are shown in table.

Of the 15 participants with positive bio-aerosol cultures, 10 had one or more CFU on the viable Andersen impactor. CFU were found on all stages of the impactor ( Figure 4). In addition, for one of the participants, one CFU was found on the polycarbonate filter located at the Andersen impactor outflow. Particle counts, measured by the APS, and matched by size with the impactor stages were used to establish the aerosol volume as a denominator. The calculated CFU count per millilitre of bio-aerosol is displayed in Figure 4. Notably, the concentration of CFU per ml of bio-aerosol was 100 to 1000-fold higher than that found in the sputum (up to 10 CFU per ml).

### Cough analysis

**Figure 5.**
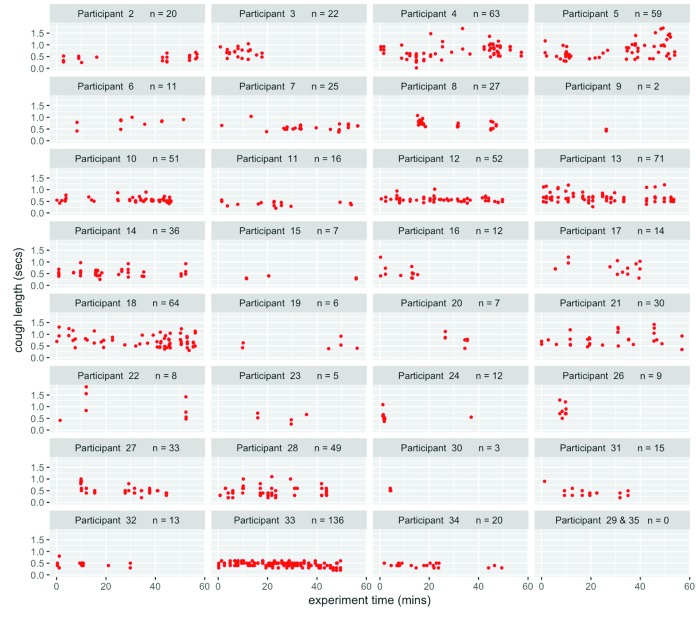
Plot of recorded coughs for each participant throughout the experiment.

To ascertain whether a unique cough signature was associated with each participant, sound recordings for the full period spent in the chamber were analysed, and the number and duration of coughs determined ( Figure 5). Sound recording was not performed for the first and 25th participants. No statistically significant correlation was detected between cough frequency and particle production (Spearmans’ rank correlation coefficient of 0.11; p=0.55) but, as mentioned before, there is an association between culturable bio-aerosol production and cough frequency (Wilcoxon rank sum test p=0.022).

### Fungal contamination

A significant percentage of fungal and bacterial (non- *Mtb*) contamination was found on both the solid 7H10 medium used in the impactor and from the filters. Likely fungal contamination was recorded on one or more of the impactor plates for 26% of participants, 6% of the gel filters, and 37% of polycarbonate filters. Bacterial contamination was identified on one or more impactor plate for 20% of participants, 3% of gel filters and 11% of polycarbonate filters. In combination, these data highlight the technical challenges stemming from the requirement for long-term incubation of environmentally exposed plates in order to enable *Mtb* CFU formation.

## Discussion

This study utilised a novel collection system to identify and quantify the *Mtb* content of environmental bio-aerosols produced by newly diagnosed but untreated TB patients. Viable impaction onto solid media and capture onto filters allowed culture of *Mtb* organisms in nearly 40% of subjects and *Mtb* DNA amplification of filtered bio-aerosol material in more than 90% of subjects in whom the assay was successfully performed. The RASC system minimized expired bio-aerosol dilution by using a small chamber volume and minimal fresh air ventilation while available bio-aerosol was maximized by the length of time spent in a confined space and by limiting the study to untreated TB patients ^21^. The sensitivity of the RASC therefore extends the earlier work of Wells and Riley ^4^ and Fennelly ^12^ by increasing the proportion of patients in which airborne *Mtb* could be isolated and sampling bio-aerosols likely to remain in the environment for a period of time. Further sensitivity improvements may be possible with an increase in the volume of exhaled air sampled.

The intriguing observation that *Mtb*-specific RD9 DNA sequences could be detected in almost all patients with the ddPCR assay raises fundamental questions as to what these DNA sequences represent. Future work is aimed at resolving whether the *Mtb* DNA signal was from DNA incorporated in viable or non-viable *Mtb* cells or, possibly, cell-free DNA. Recent studies have identified differentially culturable *Mtb* in sputum samples ^22^,^23^. Further studies exploring the increased magnitude of PCR signal compared with CFU could explore whether differentially culturable organisms such as those reported to occur in sputum ^20^, or produced *in vitro* by rifampicin treatment of starved cultures of *Mtb*, occur in patient-generated bio-aerosols.

The finding of organisms throughout the impactor stages supports the premise that *Mtb* is indeed incorporated within respirable bio-aerosols. Analysis of the CFU distribution in conjunction with the size distribution of the measured bio-aerosols demonstrates a similar order of magnitude for the *Mtb* concentration in 1–10 μm particles across all 6 stages of the Andersen impactor. This may imply that incorporation of the bacillus into the bio-aerosol is simply proportional to aerosol volume and not specific to aerosol size.

The concentration of CFU in bio-aerosol material was approximately 1–2 orders of magnitude higher than the concentration of CFU in sputum and represented a median production rate of approximately 1 CFU per minute in exhaled breath (1 CFU per 9 litres). Culturable *Mtb* isolated in cough-derived aerosols has been shown to be associated with transmission risk ^15^. The presence of culturable *Mtb* organisms in such high concentrations in respirable bio-aerosols does suggest probable host physical or immunologic control to limit *Mtb* infection becoming widespread throughout the respiratory system.

Caveats for our study include the relatively small number of participants, all with drug-sensitive TB; these findings will need to be confirmed in larger numbers and in drug-resistant TB cases. The ddPCR assay is a very sensitive and quantitative assay but we applied extremely stringent criteria to ensure a high specificity at a cost of loss of sensitivity. The culturability of *Mtb* may have been underestimated as we relied on solid media culture and occasionally lost cultures due to fungal overgrowth. We plan to augment solid media culture with more sensitive liquid capture and culture methodologies in future studies.

## Conclusions

The use of a sensitive sampling system demonstrated a high number of *Mtb* organisms in respiratory bio-aerosols. This finding highlights the great potential for breath sampling, as both a research and a clinical tool. Generating specimens by this method selects a subpopulation of *Mtb* organisms that is very likely to be phenotypically distinct. Since this subpopulation is necessarily involved in disease transmission between hosts, further investigation would be of great interest.

In the clinical setting, a refined sampling system could be used as non-invasive diagnostic test of particular benefit in sputum-scarce or sputum smear-negative patients, a group known to be responsible for a proportion of transmission ^24^. Combining breath sampling with a molecular detection test could produce a rapid point-of-care system with high sensitivity, although this may be at the expense of false positives from non-viable organisms in previously treated individuals as has been observed with GeneXpert RIF assay ^25^. Such a test may have a clinical role as a measure of infectivity in the hospital setting or, conceivably, for mass screening to identify sub-clinical cases in high burden communities.

## Data availability

The data supporting the findings reported in this study have been uploaded to OSF: https://osf.io/3kfgy/. DOI, 10.17605/OSF.IO/3KFGY ^26^.

Data are available under the terms of the Creative Commons Zero "No rights reserved" data waiver (CC0 1.0 Public domain dedication)

Dataset 1. Particle Data.

Particle data collected throughout the experiment. Recorded as a raw count of particles separated into size bins for aerodynamic diameter. The headers represent the lower limit of the size bin (in microns).

Dataset 2. Mtb Aerosol Culture.

Results for *Mtb* culture testing for multiple sampling modalities. The number corresponds to colony forming units (CFU).

Dataset 3. Mtb Aerosol ddPCR.

Results for *Mtb* ddPCR test for multiple sampling modalities. A score of 1 corresponds to a positive result, -1 a negative result and 0 is an indeterminate result.

Dataset 4. Cough Data.

Results of sound analysis to identify spontaneous coughs during the experiment.
