# Supplementary material for: Detection of Mycobacterium tuberculosis bacilli in bio-aerosols from untreated TB patients
Source: Gates Open Res. 2018 Jun 8;1:11. Originally published 2017 Nov 7. [Version 2] doi: 10.12688/gatesopenres.12758.2 (PMC5757796; doi:10.12688/gatesopenres.12758.2)
Supplement: Peer review history

# Peer review: Referee response for version 2

**DOI:** 10.21956/gatesopenres.13906.r26509

**Published:** 2018-07-09

**Authors:** Kevin P Fennelly

**Affiliations:** 1 Laboratory of Chronic Airway Infection, Pulmonary Branch, Division of Intramural Research, National Heart, Lung and Blood Institute, National Institutes of Health, Bethesda, MD, USA

Thank you for clarifying the 'CFU in volume of bio-aerosol', which was not clear to me in the original version.   This is a new concept in the bio-aerosol literature with which I am familiar.  As such, an even more thorough description of the concept in this or subsequent papers and lectures may help other readers and investigators.  It is an interesting concept that may become more important as the field evolves.

 The last sentence of the introduction might be misleading to some readers. It suggests the concept that all so-called 'infectious droplet nuclei, i.e., all airborne particles containing *M. tuberculosis *that are less than 5 microns in diameter, originate as larger (> 5 micron particles) that must undergo an evaporative process over a long period of time. Some colleagues have misinterpreted this to be consistent with the concept that a susceptible individual had to be exposed, e.g., in a household for several hours.  Our data has consistently demonstrated that the majority of particles collected immediately from cough aerosols of *M. tuberculosis *are less that 3.3 microns, consistent with being inhalable and 'immediately' infectious.

 Thank you for clarifying the discordance in the CFU counts between Table 1 and Figure 4.

 Mold contamination has been an obstacle for others attempting to isolate *M. tuberculosis *from the air.  In our first published work on cough aerosols done in Uganda ^1^, mold contamination of the solid agar plates used to collect cough aerosols was highly associated with mold counts from the ambient air.  However, we did see occasional patients who had mold in their sputum that was collected in cough aerosols.  Given the nature of the RASC, it would be interesting to use it to differentiate these sources.

 Given the differences between your ddPCR data and your culture data, it would be very interesting to use your system to study the relative proportions of viable, viable but not culturable, and non-viable bacilli.

 I hope that these comments are helpful.

I have read this submission. I believe that I have an appropriate level of expertise to confirm that it is of an acceptable scientific standard.

## References

[1] FennellyKPJones-LópezECAyakakaIKimSMenyhaHKirengaBMuchwaCJolobaMDryden-PetersonSReillyNOkweraAElliottAMSmithPGMugerwaRDEisenachKDEllnerJJ: Variability of infectious aerosols produced during coughing by patients with pulmonary tuberculosis. *Am J Respir Crit Care Med*.2012;186(5) : 10.1164/rccm.201203-0444OC 450-7 10.1164/rccm.201203-0444OC 22798319PMC3443801

# Peer review: Referee response for version 1

**DOI:** 10.21956/gatesopenres.13818.r26214

**Published:** 2018-01-17

**Authors:** Kevin P Fennelly

**Affiliations:** 1 Laboratory of Chronic Airway Infection, Pulmonary Branch, Division of Intramural Research, National Heart, Lung and Blood Institute, National Institutes of Health, Bethesda, MD, USA

For full transparency, I should first reveal that I viewed this paper through the lens of having led the development of a cough aerosol sampling system (CASS) for the collection of cough aerosols of Mycobacterium tuberculosis.  So I was very interested in comparing this new method to the CASS, as it seems complementary to rather than competing against the CASS approach. It is exciting to see other investigators in this field, and this paper describes novel findings, particularly regarding the analysis of individual aerosol particles.   Interestingly, the 43% of aerosol-positive patients based on culture in this current paper is nearly the same as the proportion of cough aerosol-positive patients (45%) in our study linking cough aerosols to new infections in household contacts ^1^.The very high (93%) proportion detected by ddPCR is interesting, and begs the question of whether the discordance with the culture results signifies a large number of dead or non-viable bacilli vs. a large number of viable but non-culturable bacilli in the aerosol.

 I would have preferred to see the aerosol CFU data per subject in a figure rather than simply written in Table 1. A simple plot of those data sorted by the magnitude of the aerosol CFU suggests that these data are log-normally distributed, as we have observed  for cough aerosols of M. tuberculosis in our cohort in Uganda ^2^. This concept may be important given our finding that household contacts exposed to index cases producing over 10 CFU of cough aerosol were most likely to be newly infected ^1^.   This distribution also reflects the variability of infectiousness and transmission of tuberculosis, as was observed by Richard  Riley and his colleagues in the 1950s ^3^ and others, including most recently by Escombe and his team ^4^ in addition to our most recent work cited above.

 An aspect of this paper with which I struggled is the result that there is 4.5x107 CFU/ml of M. tuberculosis in the ‘exhaled bioaerosol.’   Perhaps this reflects my lack of mathematics or engineering expertise, but such a result is inconsistent with the epidemiology of TB transmission.  In the absence of the ability to measure airborne concentrations of M. tuberculosis, estimates of average health care worker (HCW) exposures in hospitals from human patients was estimated to be 1.25 infectious ‘quanta’ per hour, a quanta being the amount that would cause TB disease in a guinea pig model ^3^.   In an outbreak with prolonged exposure to an untreated patient, the estimate of aerosol production was 13 infectious quanta per hour ^5^.  An even higher concentration was estimated for exposures of  HCWs during aerosol-producing exposures in a poorly ventilated unit, 250 infectious quanta per hour ^6^. Using the Wells-Riley mathematical model of airborne infection, we estimated that the risk of infection was nearly 100% for persons exposed to a theoretical source patient who produced 1000 infectious quanta per hour in a hospital room with six air changes per hour ^7^.  The result of 4.5 x 107 CFU/ml of exhaled bioaerosol is 4.5 x 1010 CFU/L.   So if a patient is breathing a reasonable 10 L/min, he would generate 4.5 x 1011 CFU per minute.  Assuming 10 minutes of sampling using the Andersen cascade impactor, this would be 4.5 x 1012 CFU, or 27 x 1012 CFU/ hour.  This value is so strikingly at odds with the data cited above that I must question whether or not there has been a possible error in the calculations.  It would have helped me to have understood this better had all these calculations been done in the supplementary material, rather than just providing the formula.   The conclusion that thte concentration of CFU in the aerosol is up to 1000-fold higher than in the sputum seems biologically implausible to me and warrants a more thorough explanation. 

 In Table 1 and the text, there are a total of 59 CFU generated by all patients, but in Figure 4 the total CFU count is  46.  I did not follow what explained that discordance.

 Figure 5 is an interesting plot, but I did not find it helpful in understanding the association between cough and aerosol production.  An X-Y plot with cough frequency on the X-axis and 2 Y-axes with particle production and culturable aerosol would seem to be more informative.

 I was surprised by the amount of fungal and bacterial contamination reported, given that the RASC system in HEPA filtered.  Did the investigators obtain fungal and routine bacterial cultures of the sputum from their participants?   In other words, might the contamination be from the cough aerosols rather than the environment?

 While I appreciate the authors’ citing our work, many other investigators, both past and present, have worked to improve our understanding of the infectiousness of TB patients using various approaches.   Papers that they may have cited include those by Escombe ^4^ ^8^, Dharmadhikari ^9^, Williams ^10^ CML and Vanden Driessche ^11^.

 In summary, the RASC is new system with great potential to improve our understanding of the infectiousness of TB patients and the aerobiology of M. tuberculosis.   I hope that my questions and concerns can help to improve this paper and the investigators’ future research.

I have read this submission. I believe that I have an appropriate level of expertise to confirm that it is of an acceptable scientific standard, however I have significant reservations, as outlined above.

## References

[1] Jones-LópezECNamuggaOMumbowaFSsebidandiMMbabaziOMoineSMboowaGFoxMPReillyNAyakakaIKimSOkweraAJolobaMFennellyKP: Cough aerosols of Mycobacterium tuberculosis predict new infection: a household contact study. *Am J Respir Crit Care Med*.2013;187(9) : 10.1164/rccm.201208-1422OC 1007-15 10.1164/rccm.201208-1422OC 23306539PMC3707366

[2] FennellyKPJones-LópezECAyakakaIKimSMenyhaHKirengaBMuchwaCJolobaMDryden-PetersonSReillyNOkweraAElliottAMSmithPGMugerwaRDEisenachKDEllnerJJ: Variability of infectious aerosols produced during coughing by patients with pulmonary tuberculosis. *Am J Respir Crit Care Med*.2012;186(5) : 10.1164/rccm.201203-0444OC 450-7 10.1164/rccm.201203-0444OC 22798319PMC3443801

[3] RILEYRLMILLSCCO'GRADYFSULTANLUWITTSTADTFSHIVPURIDN: Infectiousness of air from a tuberculosis ward. Ultraviolet irradiation of infected air: comparative infectiousness of different patients. *Am Rev Respir Dis*.1962;85: 10.1164/arrd.1962.85.4.511 511-25 10.1164/arrd.1962.85.4.511 14492300

[4] EscombeAROeserCGilmanRHNavincopaMTiconaEMartínezCCaviedesLSheenPGonzalezANoakesCMooreDAFriedlandJSEvansCA: The detection of airborne transmission of tuberculosis from HIV-infected patients, using an in vivo air sampling model. *Clin Infect Dis*.2007;44(10) : 10.1086/515397 1349-57 10.1086/515397 17443474PMC2912511

[5] NardellEAKeeganJCheneySAEtkindSC: Airborne infection. Theoretical limits of protection achievable by building ventilation. *Am Rev Respir Dis*.1991;144(2) : 10.1164/ajrccm/144.2.302 302-6 10.1164/ajrccm/144.2.302 1907115

[6] CatanzaroA: Nosocomial tuberculosis. *Am Rev Respir Dis*.1982;125(5) : 10.1164/arrd.1982.125.5.559 559-62 10.1164/arrd.1982.125.5.559 7081816

[7] FennellyKNardellE: The Relative Efficacy of Respirators and Room Ventilation in Preventing Occupational Tuberculosis. *Infection Control and Hospital Epidemiology*.1998;19(10) : 10.2307/30141420 754-759 10.2307/30141420 9801283

[8] EscombeARMooreDAGilmanRHPanWNavincopaMTiconaEMartínezCCaviedesLSheenPGonzalezANoakesCJFriedlandJSEvansCA: The infectiousness of tuberculosis patients coinfected with HIV. *PLoS Med*.2008;5(9) : 10.1371/journal.pmed.0050188 e188 10.1371/journal.pmed.0050188 18798687PMC2535657

[9] DharmadhikariASBasarabaRJVan Der WaltMLWeyerKMphahleleMVenterKJensenPAFirstMWParsonsSMcMurrayDNOrmeIMNardellEA: Natural infection of guinea pigs exposed to patients with highly drug-resistant tuberculosis. *Tuberculosis (Edinb)*.2011;91(4) : 10.1016/j.tube.2011.03.002 329-38 10.1016/j.tube.2011.03.002 21478054PMC3339040

[10] WilliamsCMCheahESMalkinJPatelHOtuJMlagaKSutherlandJSAntonioMPereraNWoltmannGHaldarPGartonNJBarerMR: Face mask sampling for the detection of Mycobacterium tuberculosis in expelled aerosols. *PLoS One*.2014;9(8) : 10.1371/journal.pone.0104921 e104921 10.1371/journal.pone.0104921 25122163PMC4133242

[11] Vanden DriesscheKMaraisBJWattenbergMMagis-EscurraCReijersMTuinmanILBoereeMJvan SoolingenDde GrootRCottonMF: The Cough Cylinder: a tool to study measures against airborne spread of (myco-) bacteria. *Int J Tuberc Lung Dis*.2013;17(1) : 10.5588/ijtld.12.0289 46-53 10.5588/ijtld.12.0289 23232004

# Response

**Published:** 2018-06-05

**Authors:** Benjamin Patterson

**Affiliations:** Columbia University Medical Center, UK

Thank you for your helpful review. We hope that our work represents an extension of the Cough Aerosol Sampling System (CASS) approach. We have addressed the points raised:

*An aspect of this paper with which I struggled is the result that there is 4.5x107 CFU/ml of M. tuberculosis in the ‘exhaled bioaerosol.’*

**The measure of CFU in volume of bioaerosol seems to be have been misunderstood. We calculated an CFU in exhaled air volume for aerosol positive participants (median 0.1 CFU/L). We also generated an approximate “concentration” of CFU in respiratory fluid per millilitre of bioaerosol and independent of exhaled air. The denominator was calculated from the known count and size of particles in the air during the sampling phase (formulae for the calculation have been added to version 2). “CFU per millilitre of bioaerosol” is quite distinct from “CFU per litre of exhaled air” (which is a far lower concentration).**

*In Table 1 and the text, there are a total of 59 CFU generated by all patients, but in Figure 4 the total CFU count is  46.  I did not follow what explained that discordance.*

**The total number of CFU captured (59) is different from the figure 4 plot (46 CFU) since the former includes all sampling devices: impactors, cyclone collectors, and various filters whereas the latter is only the result from the Andersen impactor.**

*I was surprised by the amount of fungal and bacterial contamination reported, given that the RASC system in HEPA filtered.  Did the investigators obtain fungal and routine bacterial cultures of the sputum from their participants?   In other words, might the contamination be from the cough aerosols rather than the environment?*

**Unfortunately, we do not have sputum culture for bacteria or fungi to exclude this possibility.**

# Peer review: Referee response for version 1

**DOI:** 10.21956/gatesopenres.13818.r26105

**Published:** 2017-12-04

**Authors:** Michael R Barer

**Affiliations:** 1 Department of Infection, Immunity and Inflammation, University of Leicester, Leicester, UK; 2 Department of Clinical Microbiology, University Hospitals of Leicester NHS Trust, Leicester, UK

**General Comment**

 This is a very useful initial study with the RASC system established by the authors and reported last year. A number of points should be clarified and these are listed below.

 Patterson and colleagues report an aerosol positivity rate of 77% comprising separately 43% (15/35) by culture and 71% (25/35) by PCR. These headline rates for the two different approaches are based on cumulative results from multiple collection systems and I note that Andersen sampling was was only positive for culture in 10 cases (26%) while the key collection for PCR appeared to be “open” polycarbonate filters which gave 57% (20/35) positives supplemented by “cyclone” collections introduced in later subjects 71% (5/7).

 Given the novelty of the system it is difficult to compare the positivity rates in this study with those obtained in the cough aerosol sampling system (CASS) developed by Fennelly and colleagues. Nonetheless, the authors appear to run their Andersen samplers for a total of 10 minutes at the standard rate. Assuming that no other extract was running contemporaneously (see below), their Andersen positivity appears significantly lower than the 45% positivity reported for CASS in reference 13 while the overall culture positivity of 43% is very close. The authors do not comment on our own exhaled air studies, which yielded a positivity rate of 65% by face mask sampling .

 Clearly there are significant differences between the sampling methods, notably subjects are asked to cough for CASS and were not in the mask study, but I think it is important to present these results in a manner that facilitates comparisons. There is a case for reporting all the detections against litres of air sampled by the specific method sampled.

**Specific Comments**

1.  More detail is required on the sampling protocol:
  a. How many air changes are achieved in the ten minute purge period?
  b. In the initial participant contamination phase is the chamber sealed or is there passive diffusion across the intake HEPA filters.
  c. When sampling was initiated how many extracts through samplers are running at the same time? What was the sequence of running different samplers and was this altered between subjects?
  d. Was the extract maintained at a constant overall rate during the sampling period or did this vary according to the sampler deployed? Clearly variations in dilution rate will have a profound effect on the yield of sampling
  e. Was the internal air made up by passive diffusion across the intake HEPA filters?
  f. Were temperature and humidity monitored in the sampling chamber – differences between subjects could have a profound effect on aerosols and bacterial culturability?
  g. It is excellent that specific clothing was used during sampling. Nonetheless, it is important to show that TB negative individuals exposed to the same environment as TB positive subjects for similar periods prior to sampling do not carry bacilli into the chamber.
  h. It appears that the target CO2 levels were not attained with several subjects. It would be useful to have more information on this – how many, how far away from the threshold, was this associated with poor results?
  i. Details of cyclone sampling should be given in Methods.
2. Sample analyses
  a. Quantification: Please show the formulae used to calculate volume of exhaled air per subject and CFU per ml of exhaled air.
  b. Andersen impactors: was the same medium used for direct agar impaction and filter analysis. Why was Tween 80 included and how much? Was inclusion of selective antibiotics considered?
  c. PCR: What was the estimated limit of detection?
3. Results
  a.PCR – Why are quantitative results not given? It would be useful to know if they correlate with colony counts and to get some idea of the scale of discrepancy.
  b. Culture: This seems generally clear but I don’t understand how “4.5x10 CFU/ml” was arrived at. Is this per ml expired air?
  b. Cough: The statement the culturable aerosols were associated with higher cough frequencies seems to be contradicted by the abstract. I accept that an association is not a prediction but I think this could be made clearer. Was there any association with the quantitative PCR results?
  c. P8 3ln from foot “there an” – “is” missing
4. Discussion
  a. PCR vs culture detection. I agree that that the discrepancies observed suggest the possible presence of differentially culturable/detectable bacilli and that this deserves further investigation. However, such bacilli were first reported in sputum by our group and we have also provided evidence of high numbers of bacilli supporting phage replication in our mask studies .
  b. The estimated concentration of CFU in bioaerosol calculations are intriguing. Display of the formula used is essential but I find the result plausible. Does the calculation take into account the likely reduction in bioaerosol volume prior to particle sizing?
  c. Culturable aerosol was associated with risk in reference 14 but I could not find such data in ref 15 (which is repeated as 17)

I have read this submission. I believe that I have an appropriate level of expertise to confirm that it is of an acceptable scientific standard.

## References

[1] WilliamsCMCheahESMalkinJPatelHOtuJMlagaKSutherlandJSAntonioMPereraNWoltmannGHaldarPGartonNJBarerMR: Face mask sampling for the detection of Mycobacterium tuberculosis in expelled aerosols. *PLoS One*.2014;9(8) : 10.1371/journal.pone.0104921 e104921 10.1371/journal.pone.0104921 25122163PMC4133242

[2] MukamolovaGVTurapovOMalkinJWoltmannGBarerMR: Resuscitation-promoting factors reveal an occult population of tubercle Bacilli in Sputum. *Am J Respir Crit Care Med*.2010;181(2) : 10.1164/rccm.200905-0661OC 174-80 10.1164/rccm.200905-0661OC 19875686PMC2809243

# Response

**Published:** 2018-06-05

**Authors:** Benjamin Patterson

**Affiliations:** Columbia University Medical Center, UK

Thank you for your detailed review. We have attempted to address the points you have raised.

 Sampling Protocol:

*How many air changes are achieved in the ten-minute purge period?*

**Approximately 3.6 air changes were made during the 10-minute purge phase**

*In the initial participant contamination phase is the chamber sealed or is there passive diffusion across the intake HEPA filters*

**There is small volume passive diffusion across the HEPA filters during the contamination phase.**

*When sampling was initiated how many extracts through samplers are running at the same time? What was the sequence of running different samplers and was this altered between subjects?*

**The sequence of sampling was the same as mentioned in the original paper with the exception of the addition of either one or two cyclone samplers for the final four participants.**

*Was the extract maintained at a constant overall rate during the sampling period or did this vary according to the sampler deployed? Clearly variations in dilution rate will have a profound effect on the yield of sampling*

**Once sampling began a steady state CO _2_ was typically maintained at close to the peak CO _2_ level achieved.**

*Was the internal air made up by passive diffusion across the intake HEPA filters?*

**The internal air of the chamber was a combination of diffusion across the HEPA filters and exhaled participant air.**

*Were temperature and humidity monitored in the sampling chamber – differences between subjects could have a profound effect on aerosols and bacterial culturability?*

**Temperature and humidity were measured and did not differ significantly between participants.**

****

*It is excellent that specific clothing was used during sampling. Nonetheless, it is important to show that TB negative individuals exposed to the same environment as TB positive subjects for similar periods prior to sampling do not carry bacilli into the chamber.*

**TB negative controls and empty booth controls were included and will be the subject of a future publication.**

*It appears that the target CO2 levels were not attained with several subjects. It would be useful to have more information on this – how many, how far away from the threshold, was this associated with poor results?*

**13 out of 35 participants did not reach the target 4000 PPM over ambient CO _2_. For these the mean CO _2_ attained was 3700 PPM and there was no significant difference between the bioaerosol culture positivity rate for these cases compared with those reaching the target.**

*Details of cyclone sampling should be given in Methods.*

**The cyclone samplers will be described in greater detail in a future publication.**

 Sample Analyses:

*Quantification: Please show the formulae used to calculate volume of exhaled air per subject and CFU per ml of exhaled air.*

**The formulae used for calculation of CFU in volume of air and volume of bioaerosol have been added to version 2.** 

*Andersen impactors: was the same medium used for direct agar impaction and filter analysis. Why was Tween 80 included and how much? Was inclusion of selective antibiotics considered?*

**The same medium was indeed used for impaction as for filter analysis. 0.05% (v/v) Tween80 was used (50 uL of Tween80 per 100 mL of media/buffer) to avoid clumping of bacilli. Selective PANTA (BD) antibiotic mixture was included.** 

*PCR: What was the estimated limit of detection?*

**The ddPCR assay is in development and will be the subject of on-going studies.** 

 Results:

*PCR – Why are quantitative results not given? It would be useful to know if they correlate with colony counts and to get some idea of the scale of discrepancy.*

**The ddPCR assay is in development and was felt to be insufficiently robust to give quantitative results.**

*Culture: This seems generally clear but I don’t understand how “4.5x10 CFU/ml” was arrived at. Is this per ml expired air?*

**The 4.5 x 10 CFU per ml of bioaerosol figure is the median value for all the participants of CFU count captured on all stages of the Andersen impactor per ml of bioaerosol. This is distinct from the CFU concentration in exhaled air which is given separately. The formulae for both calculations are included in version 2.**

*Cough: The statement the culturable aerosols were associated with higher cough frequencies seems to be contradicted by the abstract. I accept that an association is not a prediction but I think this could be made clearer.*

**The abstract has been amended to clarify this.**

*P8 3ln from foot “there an” – “is” missing*

**This typographical error has been corrected.**

 Discussion:

*PCR vs culture detection. I agree that that the discrepancies observed suggest the possible presence of differentially culturable/detectable bacilli and that this deserves further investigation. However, such bacilli were first reported in sputum by our group and we have also provided evidence of high numbers of bacilli supporting phage replication in our mask studies .*

**Thank you for drawing attention to this work; we have amended our references.**

*The estimated concentration of CFU in bioaerosol calculations are intriguing. Display of the formula used is essential but I find the result plausible. Does the calculation take into account the likely reduction in bioaerosol volume prior to particle sizing?*

**Particle desiccation was not corrected for in the calculation for concentration in bioaerosol. So this value reflects concentration of aged respired particles rather than concentration at the point of aerosol formation.**

*Culturable aerosol was associated with risk in reference 14 but I could not find such data in ref 15 (which is repeated as 17)*

**The reference error has been corrected.**

# Peer review: Referee response for version 1

**DOI:** 10.21956/gatesopenres.13818.r26103

**Published:** 2017-12-01

**Authors:** Nicola Mario Zetola

**Affiliations:** 1 Botswana-UPenn Partnership, University of Pennsylvania, Gaborone, Botswana; 2 School of Medicine, University of Botswana, Garborone, Botswana

Tuberculosis is now the number one global cause of mortality due to an infectious etiology. In settings hyperendemic for tuberculosis, ongoing transmission is the most important driver of epidemics. Our ability to interfere with ongoing cycles of TB transmission and acquisition is directly and negatively impacted by our limited understanding of the mechanical, biological and immunological factors driving the production of infectious aerosols by index TB cases. Until now, gaining insights regarding factors leading to tuberculosis transmission has been limited by the lack of tools to appropriately study the TB transmission process, while measuring and accounting for the large number wide variety of factors potentially contribute to it. Recently, the authors published the results of the validation of a respiratory aerosol sampling chamber (RASC) for the real-time investigation of TB transmission. The development and validation of these procedures represented a very significant progress in this field. In the present study, the authors expand on their previous work to gain further insight into the airborne face of TB transmission. Importantly, in this study the authors aim to quantify the bacillary concentration in exhaled aerosols through the use of their RASC among 35 newly diagnosed, Xpert-positive TB cases. This study represents the 1 application of RASC for the study of TB transmission among TB patients. Accordingly, the importance and significance of this initial (“pilot?”) study to the field of TB transmission cannot be overemphasized.

 The authors present a very well-written and carefully drafted manuscript. Despite the large amount and complexity of the data, the authors made a very good job presenting their results in an understandable fashion. The use of figures and diagrams is particularly helpful. Similarly, authors' interpretation of the results and discussion measured and appropriate.

 Few comments and suggestions that the authors may want to consider include:

 This reviewer understands that RASC is not, *per se*, a diagnostic tool. Nevertheless, better adherence to the STARD 2015 guidelines for the reporting of diagnostic accuracy studies will likely improve the quality and comprehensiveness of the manuscript. Some of those data may need to be included as supplementary tables/information to comply with the journal’s length restrictions.

 This study truly represents a multidisciplinary effort which borrows heavily from physics and engineering for biomedical applications. This is certainly a strength of the study. However (and despite great efforts by the authors to simplify their language), the understanding of several key concepts from physics and engineering remains difficult for researchers and clinicians (like me) with limited technical understanding of the topic. The addition of a line or 2 spelling out the meaning of certain key concepts will likely make this manuscript more easily understandable and appealing to a broader audience.

 The authors present the association of traditional predictors of infectiousness stratified and aerosol culture positivity in Table 2. It would also be useful to report such associations with the outcome treated as a continuous variable (Is there an association between clinical characteristics and particle sizes or number of CFUs? Interestingly, the 3 patients with CFU counts over 4 are HIV-negative (numbers are too small to draw conclusions though). Also, is there an association between bacilary burden in the sputum (as indicated by semi-quantitative AFB, time-to-culture positivity or Xpert signal) with aerosol culture positivity?

 Could you please provide a brief explanation for the discordant results seen in participant 4? The participant has a positive culture yet a negative ddPCR. Depending on what “N/A” means (see minor comment below), patients 5, 25, 29 and 31 may also require similar explanations. Understanding the potential reasons driving those discordances will provide further insights into the “diagnostic performance” (for lack of a better term) of RASC when using culture vs. ddPCR. This might be of particular importance if the authors are inclined to use culture positivity and a surrogate for infectiousness (which is a fairly reasonable assumption if interpreted in the context of the false positives/negatives intrinsic to the performance of the technique).

 Along the same lines, how can the authors explain the positive ddPCR in participant 35 who did not cough at all during the experiment?

 The finding that the concentration of CFUs per mL of aerosol is 100-1000 times higher than that in sputum is an important one and it may deserves to be emphasized/discussed further in the appropriate section.

 This first study looks into the usefulness of RASC in a very well-defined and “clean” population (all patients with Xpert-positive, drug-susceptible TB). The performance of RASC for the study of a more diverse population is likely to inferior to the one currently reported. This needs to be acknowledged in the limitations section. This problem might be even more important for the use of this approach for the study of TB transmission under “real life” conditions.

**Minor comments:**

 References 15 and 17 are repeated.

 References 13 and 14 are also duplicated

 Please clarify if “N/A” in Table 1 means “Not available” or “Not applicable”

 Given that RASC is hermetically sealed/closed during the procedure, the authors may want to consider distinguishing alveolar breathing from dead space in futures experiments (through plethysmography or procedures using similar principles). This is by no means a limitation of the current study but may provide important information in the future. The strong correlation between CO2 and particle production suggests that particles may be produced, primarily, in the lower airways (respiratory portion of the airways – which is the only air carrying CO2) as opposed to coming from cavities (which are, mostly, dead space) communicating with larger bronchi.

I have read this submission. I believe that I have an appropriate level of expertise to confirm that it is of an acceptable scientific standard.

# Response

**Published:** 2018-06-05

**Authors:** Benjamin Patterson

**Affiliations:** Columbia University Medical Center, UK

Thank you for you considered review. We have addressed the specific points raised:

*The authors present the association of traditional predictors of infectiousness stratified and aerosol culture positivity in Table 2. It would also be useful to report such associations with the outcome treated as a continuous variable (Is there an association between clinical characteristics and particle sizes or number of CFUs?*

**Traditional predictors of infection (sputum smear status, cavitary disease on chest radiograph, cough rate) were not correlated with aerosol culture positive treated as either binary or a continuous: this has been clarified. We agree that this may reflect the relatively small number of subjects.**

*Interestingly, the 3 patients with CFU counts over 4 are HIV-negative (numbers are too small to draw conclusions though). Also, is there an association between bacilary burden in the sputum (as indicated by semi-quantitative AFB, time-to-culture positivity or Xpert signal) with aerosol culture positivity?*

**Unfortunately, we do not have data on bacillary burden in the sputum.**

*Could you please provide a brief explanation for the discordant results seen in participant 4? The participant has a positive culture yet a negative ddPCR.*

**Although the RASC is a sensitive system the detection of a single exhaled *MTB* bacillus is presumably a low probability event and so discrepant results across sampling modalities is not surprising.**

*Along the same lines, how can the authors explain the positive ddPCR in participant 35 who did not cough at all during the experiment?*

**Agree that this is an intriguing finding. We suspect that coughing may not be necessary to produce infectious aerosol and this will be the subject of a future publication.**

*This first study looks into the usefulness of RASC in a very well-defined and “clean” population (all patients with Xpert-positive, drug-susceptible TB). The performance of RASC for the study of a more diverse population is likely to inferior to the one currently reported. This needs to be acknowledged in the limitations section. This problem might be even more important for the use of this approach for the study of TB transmission under “real life” conditions.*

**There is on-going development of the RASC and discussion of the sensitivity of the system will be detailed in future publications.**

*References 15 and 17 are repeated.*

*References 13 and 14 are also duplicated*

**References have been amended**

*Please clarify if “N/A” in Table 1 means “Not available” or “Not applicable”*

**Not available i.e. not tested**
